# The Lessons and Experiences That Can Be Learned From China in Fighting Coronavirus Disease 2019

**DOI:** 10.3389/fpubh.2020.00227

**Published:** 2020-05-27

**Authors:** Jianli Liu, Guangming Zhang, Feng Zhang, Chunjuan Song

**Affiliations:** ^1^School of Textile Science and Technology, Jiangnan University, Wuxi, China; ^2^The University of Texas Health Science Center at Houston, Houston, TX, United States; ^3^School of Biology and Basic Medical Sciences, Soochow University, Suzhou, China; ^4^Jiangsu Province Hospital and Nanjing Medical University First Affiliated Hospital, Nanjing, China

**Keywords:** COVID-19, public health emergency, lessons and experiences, prevention and control measures, wildlife protection

## Introduction

The fight against the novel coronavirus disease 2019 (COVID-19) is not only a hard battle for Chinese people but a major test of China's public health emergency management system and capacity for governance. Some weaknesses and loopholes have been exposed by the battle against the epidemic, especially the urgent need to construct a coordinated system for major public health risk analysis, evaluation, decision-making, and prevention and control (1). Holding these in mind, we summarized the following six points in the context of the hard battle against COVID-19 all Chinese people are involved in.

## Early Warning Signs Used by the Authorities

Early warning signs of the epidemic will provide a window of opportunity to contain COVID-19. To fully investigate the situation of COVID-19 at an early stage, three high-level expert groups were appointed by the National Health Commission to Wuhan. The lack of scientific data fully reported by the Health Commission of Wuhan city before Jan. 20, 2020, made it difficult to evaluate the severity and risk of COVID-19 (2). As of Jan. 20, 2020, a total of 16 briefings were reported by the Health Commission of Wuhan. However, the most important characteristic of COVID-19—infectiousness - was neglected. Five of the 16 briefings reported before Jan. 11, 2020, all claimed that “no medical worker has been infected” and “no evidence shows human to human transmission.” The full evaluation of the early warning signs is very important for the final decision on the determination of a public health emergency. The third high-level expert group appointed by the National Health Commission to fight COVID-19 publicly revealed that the disease had been transmitted between people, which is one of the important turning points of the epidemic's prevention and control, and provides a window of opportunity. The novel coronavirus is a brand-new virus for human beings. There is a process of recognition and research, which is under the laws of science. Some researchers concluded that human-to-human transmission had occurred among close contacts since the middle of December 2019 through retrospective analysis (3, 4). But the realization of the fact of human-to-human transmission came too late. So far, more than 80,000 confirmed cases and more than 4,600 deaths have been reported in 31 provincial-level regions on the Chinese mainland as well as the Xinjiang Production and Construction Corps. As of Feb. 17, 2020, China has contained more than 99 percent of the confirmed cases within its borders through lockdowns and controls, and provided a window of opportunity to contain the global spread of COVID-19. Authorities' early judgment of the epidemic supports the decision of government officials to control the epidemic before the window closes completely.

## The Response by the Government

Government officials have a crucial role in rapidly and efficiently responding to COVID-19. At the early stage of the outbreak, some early warnings had been officially published by the Health Commission of Wuhan in December of 2019. However, a sluggish response in the first several weeks of the outbreak made it too late to contain COVID-19 in the early outbreak. Some senior officials of Wuhan city and Hubei province have been heavily criticized for their handling of the crisis during the early stage of the outbreak. The senior leaders of Wuhan city and Hubei province have been adjusted based on the overall situation of prevention and control of COVID-19 in the epicenter. As of Feb. 21, a total of 620 officials in Wuhan were penalized during the hardest period of the fight against COVID-19. The National Supervisory Commission decided to dispatch an inspection group to thoroughly investigate issues related to doctor Li Wenliang on Feb. 7, 2020, who was reprimanded by police for the release of the message about the COVID-19 outbreak through social software. The senior leaders of the department of justice and prison administrative bureau of Shandong and Hubei province have been held accountable due to the surge of infections in prison. The formalism and bureaucracy have been resolutely opposed, and grass-roots cadres have devoted more energy to the frontline of COVID-19. In this crisis, the community workers, volunteers, police officers, residents, and government officials have also taken equally important responsibilities and have stepped into the front lines. A responsible and transparent attitude toward epidemic control work is the primary requirement for officials, which will boost national confidence to battle COVID-19. The Chinese government has sternly penalized officials who have mishandled COVID-19 prevention and control and stepped up resolute efforts to curb virus spread in the narrowing window of opportunity since the outbreak.

## Emergency Response Supplies

Emergency response supplies are essential to fight against the epidemic and save lives. China has well-developed hospitals, but a well-developed public health system and designated hospitals for the epidemic are limited. A severe shortage of hospital beds and equipment, protective suits, masks, and goggles cannot meet the treatment of a rapid and exponential growth of infectious patients, which is responsible for the higher mortality and transmission rates during the early stages of the outbreak. A severe shortage of personal protective equipment is a common problem globally. The finite manufacturing resources and inadequate personal protective equipment storages are responsible for the severe shortage at the early outbreak. Inadequate emergency response supplies increase the infection risk of medical workers. As of Feb. 11, a total of 1,716 Chinese medical workers had been infected on the frontlines of China's battle against COVID-19, and six medical workers had died from the virus. The health personnel and basic medical commodities of Wuhan as the epicenter were overwhelmed with the accumulated number of confirmed cases. Medical workers, especially the ones in the frontline of COVID-19 intervention and control, experienced high levels of mental health stress, which brought a greater risk of psychological distress (5). The severe shortage of detection reagents for infections and the oversight of asymptomatic infection during the incubation period outside the hospital contributed to the rapid transmission of the current outbreak (6). During the outbreak of COVID-19, the top priority shifts to protecting frontline medical workers against the most immediate threat through various sources to purchase personal protective equipment. Now, the medical supply shortage has been mitigated with work being resumed by more manufacturers after the extended Spring Festival holiday. Nineteen provincial-level regions on the Chinese mainland have paired up with 16 cities across Hubei to provide medical aid with a massive influx of equipment and supplies since Feb. 10, 2020.

## Comprehensive, Thorough, and Rigorous Intervention Measures

China has been in battle mode against the disease since Jan. 23, 2020, the eve of Lunar New Year, and launched a first-level emergency response to the COVID-19 outbreak on January 24. Extraordinary measures were taken during mass population movements at Lunar New Year, including nationwide quarantine, access restrictions of urban communities, and rural villages, the closure of schools and businesses, the suspension of flights and trains into and out of Wuhan, the suspension of public transportation, services, and entertainment industries, the extension of the holiday of the Spring Festival, and even cash rewards for informing on people who came from Hubei province (7). These social distancing measures are playing an effective role by reducing social interaction between people to curb the spread of COVID-19 (8). The country mobilized medical resources nationwide to aid Wuhan and control the epidemic. As of Feb. 19, more than 40,000 medical personnel, including military medics, have been sent to Wuhan from across the country with specialties in several disciplines, including respiratory infections, heart, and kidneys. In Feb. 2020, Wuhan had 11,000 intensive care medical staff, accounting for 10 percent of the nation's total. It usually takes no more than 2 h from the receipt of instructions to the formation of the medical team, and no more than 24 h from the time the medical team gathers to reach Wuhan city. Two designated hospitals (SARS treatment-model) for the treatment of infected patients, Huoshenshan Hospital, and Leishenshan Hospital, were built in 10 days with a total of 2,900 beds and started receiving and treating patients from Feb. 2 and Feb. 8, 2020, respectively. The number of designated hospitals in Wuhan has risen to 45, and 14 makeshift hospitals, which were converted from gyms, convention, or exhibition centers, have all been put into use. Now, all confirmed cases have received medical treatment in China. The daily number of new confirmed cases outside Hubei province has been declining for weeks. The makeshift hospitals in Wuhan city have been officially closed in succession because a large number of recovered patients were gradually discharged, and no patient had been admitted since March 1, 2020. The makeshift hospital provided sufficient beds to the surge of patients in the outbreak of the epidemic, which is a precedent in the history of humans fighting infectious diseases. The rapid construction and effective operation of the makeshift hospitals have played a very important role in the prevention and control of COVID-19. At the same time, psychological interventions for people, especially for patients and frontline medical workers, are implemented through community health services and mental-health-care institutions in some provinces and cities in China during the COVID-19 crisis (9). Comparatively speaking, China's response to COVID-19 stands in stark contrast to the 2002 SARS outbreak response (10). China has taken more comprehensive, thorough, and rigorous community-based epidemic control measures to mitigate the spread of COVID-19 than we discussed in this paper. Nation-wide community lockdown has been carried out since Jan. 23, 2020. More than four million community workers have been sticking to their posts in around 650,000 urban and rural communities to contain the epidemic and ensure the supply of people's daily necessities (8).

## Strengthen Wildlife Protection

SARS, MERS, and COVID-19 all have their origins in zoonotic viruses (11). The animal source of COVID-19 has not been confirmed yet, but early research suggests a high possibility of origin in enzootic bat viruses (12). It is not the first time an epidemic has been caused by wild animals. There have been six major epidemics. (Hendra, Nipah, SARS, MERS, Ebola, and COVID-19) from 1994 to 2020 caused by proven or suspected bat-borne viruses (13). Transboundary and emerging diseases account for a larger share of human infectious illnesses. Illegal wild animal trade has opened a Pandora's box, in which various virus are sealed. Additionally, the dietary culture, including eating game food animals such as civet, bat, snake, and pangolin, has the potential for animal-to-human transmission (14). The use of wild animal parts, such as tiger bones, bear bile, rhino horns, and pangolin scales, in traditional Chinese medicine, also provides opportunities to switch viruses to new hosts and cause human infection, which should be completely replaced with some substitutes. Now, more Chinese people are pushing to end wildlife markets. The management of China's vegetable markets (generally called by the Chinese) should also be improved. Vegetables and fruit, seafood, poultry, game meat, grain, and prepared food were also sold in the same market, which have the potential to be the origin of an epidemic. The outbreak of COVID-19 has prompted China to speed up biosecurity legislation, including the management measures of the vegetable markets. On Feb. 24, 2020, China officially imposed a total ban on trade and consumption of wild animals to protect biodiversity and to help the war against COVID-19.

## Improve Public Health Emergency Mechanism

Finally, China needs to improve the mechanism for major epidemic prevention and control and the national public health emergency management system (15). The outbreak of COVID-19 has critically challenged the public health emergency management system of China that was constructed since being hard-hit by the 2003 SARS crisis. After more than one decade's construction, it seems to be incomplete for the outbreak of COVID-19 and needs an overall improvement. How to strengthen the overall reformation of the mechanism for major epidemic prevention and control and the national public health emergency management system is a great and urgent mission for the Chinese government. China needs to strengthen legislation on the national public health emergency mechanism, with a focus on laws and regulations concerning major epidemic emergency responses, prevention and treatment, biosecurity, and wildlife protection (16). At the same time, the coordinated mechanism for scientific research, epidemic control, and treatment needs to be enhanced, with focus on virus traceability and epidemiology, pathogenic mechanisms and therapeutic targets, detection reagents and rapid screening, new drug research and development and rapid preparation, clinical risk prevention, and standard formulation (1). The protection and reward mechanism of frontline healthcare staff, as well as the consideration of mental health support for lengthy periods of a public health emergency, should be strengthened. More scientific and humanistic emergency psychological crisis interventions for people affected by an epidemic, such as SARS and COVID-19, should be improved through the establishment of professional teams. The outbreak of COVID-19 is not only a big test of China's ability to fight the epidemic but also a great test of international cooperation. After achieving positive progress, it is China's turn to help the world with material supplies, financial aid, and even more valuable clinical diagnosis and treatment experiences. Now, the COVID-19 pandemic is a common challenge for all humanity, in which countries can learn from China's efforts against COVID-19.

## Conclusion

The COVID-19 virus has been basically contained in China. On April. 8, 2020, Wuhan, the former epicenter of the coronavirus outbreak, reopened after a lockdown of 76 days amid a tight coronavirus quarantine. China fought COVID-19 for over 70 days and has been forced to shut down large areas of social and economic life to slow contagion. With the full recovery of economic and social order, how to prevent clustered infections related to imported cases with regularized epidemic containment measures in the ongoing second phase has been a new great challenge for China. Now, the situation remains grim for the rest of the world who must now curb the spread of COVID-19 that has been characterized as a global pandemic. For some countries and regions, the outbreak of COVID-19 is still in the early stage. How to prevent and control COVID-19 at a global level requires different countries tailoring their responses to their scenarios. The early lessons and experiences, such as the prompt judgment of early warning signs by authorities, the responsible and correct response by government, sufficient emergency response supplies and comprehensive, thorough, and rigorous intervention measures, have provided an example for the world in coping with the epidemic and offered experience in advancing global public health governance. China's experience is helping countries currently at the start of the COVID-19 crisis to plan their responses better.

## Author Contributions

JL conceived the study, collected the data, and drafted the manuscript. GZ contributed to the design of the study. FZ and CS coordinated the study tasks and helped draft the manuscript. All authors gave final approval for publication.

## Conflict of Interest

The authors declare that the research was conducted in the absence of any commercial or financial relationships that could be construed as a potential conflict of interest.

## Abbreviations

COVID-19: novel coronavirus.
